# Translating clinical trial results into personalized recommendations by considering multiple outcomes and subjective views

**DOI:** 10.1038/s41746-019-0156-3

**Published:** 2019-08-21

**Authors:** Noa Dagan, Chandra J. Cohen-Stavi, Meytal Avgil Tsadok, Morton Leibowitz, Moshe Hoshen, Tomas Karpati, Amichay Akriv, Ilan Gofer, Harel Gilutz, Eduardo Podjarny, Eitan Bachmat, Ran D. Balicer

**Affiliations:** 1 Clalit Research Institute, Clalit Health Services, Tel Aviv, Israel; 2 Computer Science Department, Ben Gurion University of the Negev, Be’er Sheba, Israel; 3 Public Health Department, Ben Gurion University of the Negev, Be’er Sheba, Israel; 4 ADAM Institute of High Blood Pressure, Clalit Health Services, Hertzlia, Israel

**Keywords:** Preventive medicine, Databases

## Abstract

Currently, clinicians rely mostly on population-level treatment effects from RCTs, usually considering the treatment's benefits. This study proposes a process, focused on practical usability, for translating RCT data into personalized treatment recommendations that weighs benefits against harms and integrates subjective perceptions of relative severity. Intensive blood pressure treatment (IBPT) was selected as the test case to demonstrate the suggested process, which was divided into three phases: (1) Prediction models were developed using the Systolic Blood-Pressure Intervention Trial (SPRINT) data for benefits and adverse events of IBPT. The models were externally validated using retrospective Clalit Health Services (CHS) data; (2) Predicted risk reductions and increases from these models were used to create a yes/no IBPT recommendation by calculating a severity-weighted benefit-to-harm ratio; (3) Analysis outputs were summarized in a decision support tool. Based on the individual benefit-to-harm ratios, 62 and 84% of the SPRINT and CHS populations, respectively, would theoretically be recommended IBPT. The original SPRINT trial results of significant decrease in cardiovascular outcomes following IBPT persisted only in the group that received a “yes-treatment” recommendation by the suggested process, while the rate of serious adverse events was slightly higher in the "no-treatment" recommendation group. This process can be used to translate RCT data into individualized recommendations by identifying patients for whom the treatment’s benefits outweigh the harms, while considering subjective views of perceived severity of the different outcomes. The proposed approach emphasizes clinical practicality by mimicking physicians’ clinical decision-making process and integrating all recommendation outputs into a usable decision support tool.

## Introduction

Most decision support tools or risk calculators that physicians use to translate empirical trial evidence into useful clinical information for individual patients do not consider adverse events, nor do they allow for the integration of subjective input. While evidence-based medicine is well established with the integration of clinical trial research into medical knowledge and guidelines, there is increasing acknowledgement of the shortcomings of these data for individualized treatment decisions.^1,2^ Empirical evidence is still mostly reported by clinical trials and meta-analyses as population-based hazard-ratios and numbers-needed-to-treat (NNT),^3,4^ even though these group averages can change when considering individual patient characteristics and different baseline risks.^5–7^ Additionally, it is not clear how to best engage patients and integrate their preferences into clinical decisions, particularly given that subjective preferences can be at odds with evidence-based medicine.^1,2,8^

Risk models have been developed to calculate patients’ individual risks in a variety of medical fields, but most models are focused on a specific outcome^9,10^ or a composite of several outcomes,^7,11,12^ which are often not reflective of the multiple separate effects of many interventions. Many risk prediction models do not account for the trade-offs between potential benefits and harms (adverse events),^10^ and the few that do take harms into account have used a single negative treatment consequence or an overall estimation of negative effects.^4,6,13,14^ Furthermore, patient engagement and preferences are largely not actively incorporated into this weighing of benefits and harms.^15^

The uptake of decision support systems and risk calculators in clinical practice is also limited since not enough focus is placed on presenting them in a transparent and clinically-usable format that physicians can use and trust.^16^ The presentation of clinically useful outputs from analytically rigorous prediction models is critical for both physician appraisal and in the ability to engage in conversation with patients.^16–18^ The potential exists to enhance physician-patient treatment dialog through clinical decision support tools,^7^ and is something that is desired by physicians.^19^

In this study, we present an approach focused on practical usability that translates clinical trial results into individualized treatment recommendations by considering both personalized benefits and harms, and integrating them with subjective perceptions of relative severity. We also aimed to design a clinically-relevant decision support tool concept that presents individualized risk estimates, which includes a built-in ability to enter patient or physician preferences, and presents the final treatment recommendation. The suggested process is demonstrated using data from a clinical trial that examined intensive systolic blood pressure treatment.

## Results

### Study population

The SPRINT population included 9360 individuals and the CHS population consisted of 88,374 individuals. A total of 1.2% of the SPRINT participants and 1.4% of the CHS members had one or more missing predictor values that were imputed. The baseline characteristics of the SPRINT and CHS populations are detailed in Table 1. The clinical trial SPRINT population and the routine practice CHS population differed in their composition, with a larger proportion of younger adults (67.9 ± 9.4 vs. 71.7 ± 10.7) and males (64.4 vs. 52.8%) in the SPRINT population. The CHS population included a small proportion of black race individuals (mostly Ethiopian) and had a higher rate of prevalent cardiovascular disease at baseline (Table 1).

**Table 1:** Study populations by the covariates used in the models

	SPRINT population	CHS population
Characteristics^a^	*N* = 9360	*N* = 88,374
Age, years		
Mean ± SD	67.9 ± 9.4	71.7 ± 10.7
Missing, *n* (%)	0 (0.0%)	0 (0.0%)
Sex		
Female, *n* (%)	3,331 (35.6%)	41,713 (47.2%)
Male, *n* (%)	6,029 (64.4%)	46,661 (52.8%)
Missing, *n* (%)	0 (0.0%)	0 (0.0%)
Black race		
No, *n* (%)	6,414 (68.5%)	87,456 (99.0%)
Yes, *n* (%)	2,946 (31.5%)	918 (1.0%)
Missing, *n* (%)	0 (0.0%)	0 (0.0%)
BMI^b^, kg/m^2^		
Mean ± SD	29.9 ± 5.8	28.4 ± 5.0
Missing, *n* (%)	77 (0.8%)	260 (0.3%)
Smoking category		
Never, *n* (%)	4,122 (44.0%)	56,790 (64.3%)
Former, *n* (%)	3,973 (42.4%)	17,472 (19.8%)
Current, *n* (%)	1,239 (13.2%)	13,166 (14.9%)
Missing, *n* (%)	26 (0.3%)	946 (1.1%)
Clinical/subclinical CVD		
No, *n* (%)	7,483 (79.9%)	58,526 (66.2%)
Yes, *n* (%)	1,877 (20.1%)	29,848 (33.8%)
Missing, *n* (%)	0 (0.0%)	0 (0.0%)
Systolic BP, mmHg		
Mean ± SD	139.7 ± 15.6	139.9 ± 10.0
Missing, *n* (%)	0 (0.0%)	0 (0.0%)
BP medication types		
0–1, *n* (%)	3,635 (38.8%)	42,576 (48.2%)
2–3, *n* (%)	5,211 (55.7%)	41,842 (47.3%)
≥4, *n* (%)	514 (5.5%)	3,956 (4.5%)
Missing, n (%)	0 (0.0%)	0 (0.0%)
eGFR, mL/min/1.73 m^2^		
Mean ± SD	71.7 ± 20.6	78.2 ± 21.1
Missing, *n* (%)	37 (0.4%)	127 (0.1%)
Total cholesterol, mg/dL		
Mean ± SD	190.1 ± 41.2	188.0 ± 38.3
Missing, *n* (%)	38 (0.4%)	134 (0.2%)
HDL, mg/dL		
Mean ± SD	52.9 ± 14.5	50.4 ± 13.4
Missing, *n* (%)	38 (0.4%)	146 (0.2%)

*SPRINT* systolic blood pressure intervention trial, *CHS* Clalit Health Services, *SD* standard deviation, *BMI* body mass index, kg/m^2^, *CVD* cardiovascular disease, *BP* blood pressure, *eGFR* estimated glomerular filtration rate, mL/min/1.73 m^2^, *HDL* high-density lipoprotein

^a^Variables were taken from the baseline characteristics of the SPRINT population and from the available EHR information prior to January 1st, 2013 for the CHS population

^b^Body mass index was measured as the weight in kilograms divided by the square of the height in meters

### Development and external validation of risk prediction models

The models included an average of 6.8 predictor variables. Further details on the variables included in each prediction model and their coefficients are in the supplemental material (Supplementary Table 1). The discrimination and overall calibration of the prediction models in the SPRINT population are presented in Supplementary Table 2 for both final and out-of-sample models, which yielded similar performance. The AUC of the final eight models for the SPRINT population ranged between 67.8 and 77.5%. The observed-to-predicted ratios of models ranged from 0.45 to 1.17 for cardiovascular outcomes and from 1.07 to 1.47 for adverse events (Supplementary Table 2). The cardiovascular death model demonstrated a tendency to over-predict the risk, while the hypotension model tended toward under estimating the risk. Calibration plots (Supplementary Fig. 1) and observed-to-predicted ratios by deciles (Supplementary Table 3a, b) are presented in the supplement. The AUC measures in the external validation of the CHS population were similar to the SPRINT AUC’s (Supplementary Table 2) and had an increasing number of events across deciles (Supplementary Table 3a, b), indicating consistent discrimination ability in an external population. Most models in the CHS population presented observed-to-predicted ratios close to one, indicating relatively good calibration (Supplementary Table 2). The average observed-to-predicted ratio of the hypotension model was around 0.5, while the stroke and acute kidney injury models presented average observed-to-predicted ratios of 1.5–1.6.

### Treatment recommendation development and testing

**Fig. 1:**
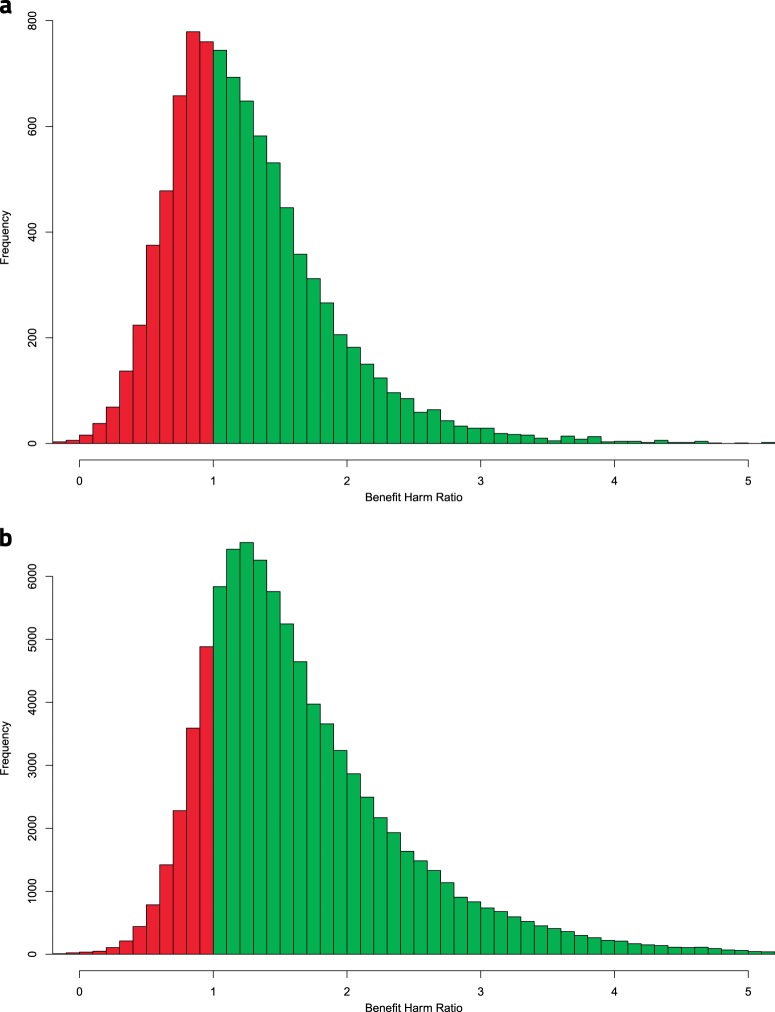
Distributions of the benefit-harm ratios. **a** Histogram of benefit-harm ratio distribution among the SPRINT population. **b** Histogram of benefit-harm ratio distribution among the CHS population. SPRINT, Systolic Blood Pressure Intervention Trial; CHS, Clalit Health Services

The severity weights that were assigned to the different outcomes were relatively consistent between the ranking physicians, with a maximum standard deviation of 1.56 (Supplementary Table 4). After applying the average severity weights, a total of 62.1 and 84.3% of the SPRINT and CHS populations, respectively, were assigned a recommendation for intensive treatment (Supplementary Table 5). The distributions of the benefit-harm ratios and treatment recommendations for both populations are presented in Fig. 1.

The group that received a recommendation for intensive treatment was relatively older (69.7 ± 9.1 vs. 64.9 ± 9.2 years in the SPRINT population and 72.5 ± 10.5 vs. 67.2 ± 10.8 years in the CHS population) and consisted of a higher percent of males (73.8 vs. 49.1% in the SPRINT population, and 55.6 vs. 37.6% in the CHS population) than the non-intensive treatment recommendation group (Supplementary Table 5). Additionally, the intensive treatment recommendation group had more cardiovascular disease at baseline (27.9 vs. 7.2% in the SPRINT population, and 38.2 vs. 9.9% in the CHS population) and relatively higher estimated glomerular filtration rate (76.9 ± 18.0 vs. 63.3 ± 21.8 mL/min/1.73 m² in the SPRINT population, and 80.6 ± 19.4 vs. 65.7 ± 25.1 in the CHS population).

The sensitivity analysis of the severity weights by different physicians demonstrated that most physicians yielded rates of ‘yes’ recommendations in the range of 50–70% (Supplementary Table 6a). One physician gave relatively low severity weights to the adverse events, resulting in ‘yes’ treatment recommendation for 89.9% of the SPRINT population, while another physician gave relatively high weights to the adverse events, resulting in a ‘yes’ recommendation for 39.8% of SPRINT patients. The additional sensitivity analysis on agreement in the resulting treatment recommendation across different sets of severity weightings showed complete agreement on the treatment recommendation for about 50% of the patients (Supplementary Table 6b).

When evaluating the effects of intensive blood pressure lowering in each recommendation group, the SPRINT’s results of a significant decrease in the composite primary cardiovascular outcome and all-cause mortality were only repeated in the intensive treatment recommendation group (hazard ratios of 0.68 and 0.65, respectively; *P* ≤ 0.001) (Table 2). The intensive treatment was found to increase the rate of the serious adverse events’ composite outcome by 45 and 41% in the non-intensive and intensive treatment recommendation groups, respectively. The two recommendation groups had similar systolic blood pressure measurements during follow-up, with the intensive treatment assigned patients from the trial within each recommendation group reaching their systolic target (121–122 mmHg). These results were consistent when evaluated using the out-of-sample models (Supplementary Table 7).

**Table 2:** Evaluation of the SPRINT outcomes within each recommendation group for validating the treatment recommendation^a^

	SPRINT randomization		Total SPRINT population	Intensive treatment recommendation group	Non-intensive treatment recommendation group
Number of participants, *n*	Actual intensive		4,677	2,916	1761
	Actual non-intensive		4,683	2,899	1784
Mean systolic BP^b^, mmHg	Actual intensive		121.01	120.68	121.55
	Actual non-intensive		135.28	134.99	135.74
Primary composite cardiovascular outcome, per year	Actual intensive		1.65	1.78	1.45
	Actual non-intensive		2.19	2.60	1.55
		Ratio (95% CI)	0.75 (0.64–0.89)	0.68 (0.56–0.84)	0.93 (0.69–1.26)
		*P*-value	0.00092	0.00022	0.64769
All-cause mortality, per year	Actual intensive		1.03	1.01	1.05
	Actual non-intensive		1.40	1.56	1.14
		Ratio (95% CI)	0.74 (0.60–0.91)	0.65 (0.50–0.84)	0.92 (0.65–1.31)
		*P*-value	0.00355	0.00105	0.64574
Serious adverse event composite outcome, per year	Actual intensive		2.89	2.59	3.38
	Actual non-intensive		2.03	1.84	2.32
		Ratio (95% CI)	1.42 (1.22–1.65)	1.41 (1.15–1.72)	1.45 (1.16–1.82)
		*P*-value	< 0.00001	0.00092	0.00123

*SPRINT* systolic blood pressure intervention trial, *BP* blood pressure, *CI* confidence interval

^a^Values of outcomes are presented as yearly incidence

^b^Using the last three measurements of each participant during follow-up

### Clinical decision support tool interface

**Fig. 2:**
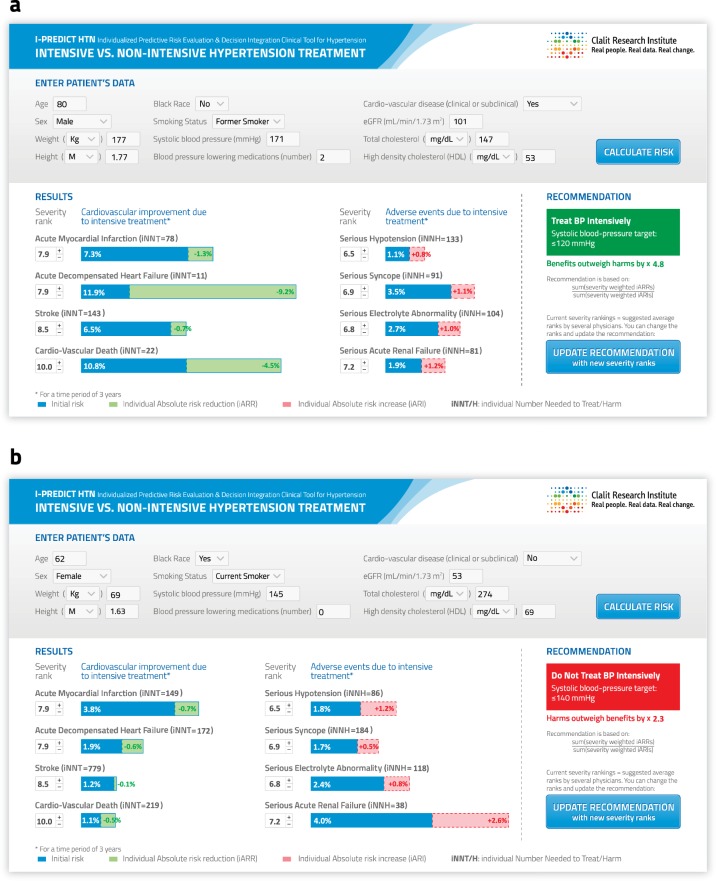
Intensive treatment decision support tool interface. **a** Example of a patient who would receive a recommendation for intensive treatment. **b** Example of a patient who would receive a recommendation for non-intensive treatment. Legend: Blue represents initial risk, green represents iARR, and pink represents iARI. eGFR, estimated glomerular filtration rate; kg, kilograms; m, meters; iNNT, individual number needed to treat; iNNH, individual number needed to harm

The tool’s interface is presented in Fig. 2. The first section, used for entering patients’ characteristics, would be filled automatically when the tool is implemented within an EHR system. The second section presents the individualized baseline risk for each of the cardiovascular outcomes and adverse events (based on the competing-risk models), along with the absolute decrease or increase in risk (iARR/iARI) due to intensive blood pressure lowering (based on the cause-specific models). This section also presents the severity weights that were used for the calculation of the final treatment recommendation. These weights can be adjusted by physicians, with or without their patients’ input, since judgements concerning the severity of outcomes may differ among clinicians and patients. The third section presents the final treatment recommendation, with the extent to which the indicated recommendation outweighed the alternative recommendation (a number representing the strength of the recommendation as a proxy of the benefit-harm ratio to one).

## Discussion

This study presents an approach and clinical decision support tool concept that helps to address the challenge of determining individual treatment recommendations from trial evidence. Whereas previous studies have dealt separately with various aspects of this challenge, the current study developed a comprehensive practice-focused analytic process that formalized and quantified what physicians do intuitively in treatment decision-making—consider potential benefits, harms, and subjective preferences. In addition, this study suggests a way to reflect these outputs transparently via a clinical decision support tool.

Increased attention has shifted the focus of treatment decision-making toward individualizing decisions,^20^ because many clinical trials, including the SPRINT study, conclude that treatment is significantly better than the alternative for the average trial patient; but this does not account for benefit-harm trade-offs for individual patients.

We demonstrate that in the SPRINT test case, the recommendation for intensive treatment may not be beneficial for all trial participants, but potentially only for 62% of them. It seems that the proposed process can distinguish those patients for whom the benefits of intensive blood pressure treatment outweighed potential harms from those for whom the harms outweighed the benefit (and for which benefits did not reach statistical significance). Furthermore, the individuals who would be recommended intensive treatment based on the proposed decision support tool would typically benefit more and suffer fewer negative consequences than the overall average reported in the SPRINT results.

Similar to the current study, prior research has accounted for benefit harm trade-offs of intensive blood pressure treatment.^12,21^ However, previous studies developing individualized prediction models for cardiovascular events have predominantly used a single or composite outcome or relied on population-level estimates to evaluate harms while estimating individualized benefits.^11–14,21^ Most of these studies conclude with a formalized prediction model as the final output, and almost none of them address the way in which clinicians will understand and use the analytic outputs in practice. There are decision support tools to demonstrate the effects of cardiovascular risk factors, which have created attractive interfaces to illustrate both the baseline risk and treatment effects, but they only focus on single or composite outcomes, and do not consider the adverse events.^10,22–24^ One of the stated reasons for this is that the weight of adverse events may be subjective.^10^ Our proposed interface has overcome this challenge by formalizing the combination of objective measures of risk with subjective views of severity.

Our decision to develop risk prediction models for eight separate outcomes that could be ranked for severity (rather than composite outcomes) was guided by the need to identify building blocks of patient-specific information that can be easily assimilated by clinicians and explained to patients. In contrast to using a net benefit method, in which the physician needs to determine an exchange rate between the positive and negative outcomes,^25^ the outcome specific severity rankings in our approach may be more intuitive and easily communicated to patients. We found that physicians had difficulty in assigning a severity score to a composite of different outcomes, while separating the outcomes allowed for consideration of each outcome separately. The development of models both for multiple individual positive and negative consequences helped to overcome the limitation of the net benefit method that can only account for a single positive and a single negative outcome. This approach allows for the integration of subjective perception of risk into the recommendation even when the composite of either the positive or negative outcomes include events of very different nature. The multiple risks could then be weighed on both sides of the scale, thereby allowing physicians to reach a final yes/no treatment decision that is aligned with “primum non nocere”.

The discrimination ability of the models in the external validation CHS population was comparable to that of the models developed and trained on the clinical trial population, as well as similar to the performance of the individual risk models developed in previous research.^12^ There were some discrepancies in the calibration results within the CHS population, with some overestimation of risk and some underestimation of risk, potentially due to an inability to identify all events within the CHS EHR data. The similar discriminatory performance across the two study populations suggests that these models are transferrable between populations, albeit, with the need to recalibrate when applied to new populations.^26^ The proposed process and tool in the current study yielded a recommendation for intensive blood pressure lowering in 62% of the SPRINT population, compared to 84% in the CHS population. This difference was expected since the first population represented individuals selected for a clinical trial, while the second represents a more general population.

When translating across populations and contexts, there are also considerable variations in perspectives of the importance of potential benefits and risks.^27^ The sensitivity analysis conducted to examine the impact of varying sets of physicians’ severity weights, showed that physicians with different views can affect the final recommendation result. Yet, for 50% of the patient population, there was consensus regarding the recommendation despite the variance in the severity weightings. For the rest of the patients, whose benefit-harm ratios were closer to one and less inclined to one direction, individual preferences (physician or patient) may be a more prominent factor in the final treatment decision. This illustrates that the tool offers support to decision-making rather than replacing human clinical judgment, through balancing the integration of empirically derived individualized evidence with built-in flexibility for shared decision making in clinical practice.

A valid methodology for reaching an individualized, evidence-based treatment decision, however, is not enough to assure implementation in clinical practice. The transformation of theory to practice depends on the usability and alignment of analytic outputs with clinicians’ perceptions and preferences.^16,17^ Accordingly, the tool’s interface has been designed to integrate both graphical illustrations and numerical representation of the individualized baseline risks and estimated treatment effects, along with a final treatment recommendation and its strength (i.e., the magnitude of the benefit-to-harm ratio). Two different measures of risk were included because they have been found to be understandable and interpretable for patients.^2,28^ Physicians can, therefore, determine whether specific outcomes or adverse event risks, or the final recommendation (using the suggested severity ranks or adapting them) are most informative for the treatment decision. Beyond just providing a risk score or a yes/no treatment recommendation, it is beneficial to include outputs such as the strength of the benefit-harm ratio, which is particularly important among patients for whom the treatment decision does not clearly incline to one direction (i.e., who have a benefit-harm ratio close to 1).

This study had notable limitations to consider. First, the timeline of the SPRINT trial allowed an evaluation of the absolute risks for a relatively short time period of three years. A longer follow-up period of five or ten years would be preferable for the iARR/iARI results, but likely would not have changed the final treatment recommendation (since the benefit-harm ratio is not expected to change dramatically over time). Second, the limited sample size of the SPRINT trial posed a challenge in stabilizing the coefficients of the prediction models thus needing to train the model using multiple bootstraps.

Importantly, truly individualized treatment effects are not ascertainable due to the lack of the ability to determine the counterfactual and to observe all relevant factors.^29^ As in other studies, the individualized estimates in the current study are limited to approximations of conditional average effects for patients similar to the individual patient. Related to data measurement limitations, in the translation of clinical trial data to individualized EHR-based risk for an external population, there can be discrepancies between the variable measurements used in the trials and the variable measurements found in an existing EHR database. The potential for these differences in measurements emphasizes the importance of comparing the performance of the developed models in the clinical trial and the external populations, which in the current study yielded relatively consistent performance. Furthermore, although we externally validated the prediction models, we were not able to validate the SPRINT results or the final treatment recommendation in the CHS population because the real-world context does not confer randomization to treatment. Nonetheless, since the risk models were externally validated, and their outputs translate directly to the treatment recommendation, the recommendation is likely to be valid in the CHS population. Finally, we have not yet evaluated the tool in a clinical setting, but we have demonstrated feasibility and the potential benefits.

Following an approach such as the one proposed in this study would allow for the translation of clinical trial data into an individualized clinical decision support tool that could potentially be replicated for many types of individualized dichotomous clinical decisions (e.g., medication initiation, surgical procedures, and diagnostic procedures). EHR systems provide a suitable setting for the implementation of this kind of tool, presenting physicians with an instant summary of the relevant clinical trials tailored to the characteristics of their patients. The tool can be automatically presented in the EHR only for patients who meet the inclusion criteria of the relevant trial. Rather than being an added burden to the patient visit, such a tool has the potential to facilitate the physician’s treatment decision-making and patient engagement by computing what is missing in practice: estimated individualized benefits and harms based on clinical trial evidence, that can be integrated with subjective perception of severity and combined into a single recommendation.

## Methods

### Setting

In order to demonstrate the suggested process of translating clinical trial results into individualized treatment recommendations, we used data that was made available from the Systolic Blood Pressure Intervention Trial (SPRINT)^30^ randomized clinical trial, whose methods and results have been previously described.^31,32^ This trial compared intensive management of systolic blood pressure targeting <120 mmHg with standard management targeting < 140 mmHg in patients with hypertension and high cardiovascular risk and no diabetes. The trial was stopped early because the average rate of cardiovascular events and all-cause mortality were significantly lower with intensive treatment.

Electronic health record (EHR) data from Clalit Health Services (CHS) members were used for external validation of analytic outputs which were developed using the SPRINT population as part of this study. CHS is an integrated payer/provider health care organization that provides primary, specialty, and inpatient healthcare to over half of the population in Israel (close to 4.5 million people), where there is universal healthcare coverage. All relevant patient data were extracted from the central data warehouse collating this information from inpatient, outpatient, laboratory, pharmacy, and other clinical settings in CHS.

This study was conducted, in part, using SPRINT-POP Research Materials obtained from the National Heart Lung and Blood Institute (NHLBI) Biologic Specimen and Data Repository Information Coordinating Centre, and does not necessarily reflect the opinions or views of the SPRINT-POP or the NHLBI. The study received approval from the Institutional Review Board Committee (IRBC) of Meir Hospital of CHS. Participants from the SPRINT gave written informed consent as part of the clinical trial. For the retrospective analysis using CHS data, the IRBC approval included an exemption regarding obtaining written informed consent.

### Study design

The study was divided into three phases: (1) develop models of individualized risk estimations for multiple positive and negative outcomes; (2) translate these predictions into a single treatment recommendation; and (3) integrate them into a clinically-relevant tool concept to support practicing physicians' decision making. In the first phase, eight prediction models of benefits and harms related to intensive blood pressure lowering were developed based on SPRINT data, which was collected between 2010 and 2015 (median follow-up period of 3.26 years) with baseline characteristics defined at the study entry date for each participant. These models were externally validated for their ability to predict outcomes in a 3-year period (between 2013 and 2015) in the CHS population, with baseline characteristics as of the index date, defined as January 1, 2013. The risk estimates from the eight models were weighted and consolidated into an intensive vs. non-intensive treatment recommendation, which was tested against the SPRINT randomization. Finally, a clinical decision support tool was designed to incorporate the analytic outputs while also accounting for the practicing physician’s needs during a patient visit.

### Study population

The SPRINT population used for this study has been described elsewhere.^31,32^ For the prediction models’ external validation, CHS members who met the same inclusion and exclusion criteria as in the SPRINT study were included. The CHS cohort of patients had a diagnosis of hypertension and two elevated blood pressure measurements as of the index date. The SPRINT's inclusion and exclusion criteria that could be assessed from the EHR data were applied (Supplementary Table 8).

### Development and external validation of risk prediction models

Using the data from the SPRINT population, four prediction models were developed for cardiovascular outcomes (treatment's benefits) that are commonly considered to be associated with high blood pressure: myocardial infarction, stroke, acute decompensated heart failure, and cardiovascular death. Additionally, four prediction models were developed for adverse events (treatment's harms) that were reported by the SPRINT study to occur more with intensive blood pressure treatment: hypotension, syncope, electrolyte abnormality, and acute kidney injury. For both outcomes and adverse events, only non-composite outcomes were selected.

The specific type of prediction algorithm used to model any benefit or harm, as well as methods of variable selection and the approach of addressing missing data, can vary by the specific implementation. In this demonstration, all models were developed using Cox-proportional hazard multivariate regression. Due to the size limitation of the data set in this case, predictors for each model were selected based on backwards selection, from a list of 11 baseline patient characteristics (Supplementary Table 1), which are likely to be available in clinical practice and are widely accepted as clinically-related to at least one of the outcomes.^6^ Missing predictor values were imputed using multiple imputation functions by Van Buuren et al.^33^ to create 5 imputed data sets.^34^ Further details regarding aspects of the multiple imputation and variable selection methods are described in the supplement (Supplementary Description 1).

The classic approach to randomly split the population into training and test sets was not feasible, as some of the outcomes were rare and their random distribution between sets greatly affected the models’ coefficients. To overcome this, we fitted each model using 1250 bootstraps (250 from each imputed data set) on the entire SPRINT population, drawn randomly with replacement.^35^ The coefficients from each bootstrap were averaged to create stable coefficients for the final eight models, and their variance was calculated using Rubin’s rules for variance estimation in multiple imputed data sets.^36,37^

Each model training in each bootstrap was evaluated twice: first using a simple Cox regression (cause-specific) in order to obtain coefficients that represent the true nature of the treatment effect on each outcome;^38^ and second using a weighted Cox by the Fine-Gray method for competing risks in order to receive a valid estimation of the baseline risk (considering death from any cause as the competing event for each outcome).^39^ In addition, to assure lack of overfitting, we calculated a set of out-of-sample models for each SPRINT participant (for both the cause-specific and competing risk models), using the average coefficients of all bootstraps in which he or she did not participate (about one third of the bootstraps).^40^

The models' performance in predicting the baseline risk within the SPRINT population was evaluated for discrimination by examining area under the receiver-operator-characteristic curves (AUC) and for calibration. The performance was evaluated using the competing risk coefficients of the final models and out-of-sample models.^26^ The AUCs and their variances were calculated using 1,250 bootstraps (with 250 from each imputed data set). To create complete independence between stages, these were newly created bootstraps, not those used for the model fitting stage. Calibration was assessed by comparing average predicted risks with observed percentages of events, stratified by deciles of risk.^26^ This analysis was done separately in each imputed data set and then averaged.

External validation for the performance^41^ of seven of the prediction models in predicting the baseline risk were conducted in the CHS population (data on cause of death was not available, and therefore, the cardiovascular death model was not validated). Details on variable definitions from the CHS EHR data are described in Supplementary Table 9. Three-year risks for outcomes and adverse events were calculated retrospectively as of the index date and compared to the actual event occurrence until the end of 2015 (when the SPRINT results were published). Missing values for one or more of the predictors in the CHS population were imputed using multiple imputation in the same manner that was used for the SPRINT population.

### Treatment recommendation development and testing

The translation of the risk estimates to a treatment recommendation was intended to mimic the clinical decision-making process undertaken by physicians, considering both the potential magnitude of the estimated benefits and harms, as well as their relative weight in terms of severity. The magnitude of predicted benefits of intensive blood pressure lowering was assessed by entering each patient’s individual characteristics into the cause-specific cardiovascular outcome models and calculating three-year individual absolute-risk-reduction (iARR) measures. On-treatment risk (intensive treatment variable set to yes) was subtracted from off-treatment risk for each specified outcome (intensive treatment variable set to no).^4^ This process was repeated for each adverse event by subtracting the off-treatment risk from the on-treatment risk, thus creating four individual-absolute-risk-increase (iARI) measures.

The severity of each cardiovascular outcome and serious adverse event was considered on a scale ranging from 0–10, through a blinded independent evaluation by 12 physicians with various relevant clinical backgrounds: general physicians, family physicians, internal medicine experts, cardiologists, nephrologists, and public health experts (two of each). The physicians were asked to assign a severity weight to each of the eight model outcomes and read the SPRINT definition of serious adverse events prior to their evaluation.^31^ The severity weights by each physician were averaged to create a final severity weight (SW) for each outcome.

To determine the treatment recommendation for the individual patient, a benefit-harm ratio was calculated by dividing the sum of weighted benefits (positive effects' iARR) by the sum of the weighted harms (adverse events' iARI): (∑SW_j_∙iARR_j_)/(∑SW_k_∙iARI_k_). A ratio >1 indicated that the benefits outweighed the harms, thus favoring intensive treatment.

To assess the impact of the severity weights on the overall distribution of the final treatment recommendation, sensitivity analyses were conducted using the sets of severity weights from each of the 12 individual physicians separately. As a further test of the effect of the severity weightings on the treatment recommendation, the proportion of patients for whom there was agreement in the resulting recommendation across the 12 physicians’ severity weights was examined.

The treatment recommendation was tested using the SPRINT’s randomization to evaluate the intensive treatment effect within each recommendation group (yes/no intensive treatment). We evaluated the treatment’s effect on the annual incidence of all-cause mortality, the SPRINT's primary composite cardiovascular outcome, and a composite of the four serious adverse events. These outcomes were chosen to allow comparison to the original SPRINT results. To overcome the limitation of testing the recommendation in the same population in which the models were developed, this evaluation was performed using predictions of both the final and out-of-sample models. The analysis was carried out only on the first imputed data set (since each participant had to have one final recommendation). The treatment recommendation could not be externally validated, since the CHS population was not randomized to the two treatment groups.

### Clinical decision support tool interface

A clinical decision support tool was designed aiming to achieve practical utility and transparency of the analytic process that led to the final recommendation. Particular attention was given to the visual presentation and the type of measurements presented. Given different physicians’ preferences for measures of risk estimation, iARRs and iARIs were incorporated, as well as, individual-number-needed-to-treat (iNNT) for each cardiovascular outcome and individual-number-needed-to-harm (iNNH) for each adverse event. The tool’s interface was developed in iterations through receiving feedback from practicing clinicians of different specialties until a consensus was reached on willingness to implement the tool in their practice.

### Statistical analysis

All analyses were conducted using R, CRAN version 3.5.2. *P*-values < 0.05 were considered significant (two-sided tests were used). Specific analyses for each step of the process have been outlined in the respective sections above.

### Reporting summary

Further information on research design is available in the Nature Research Reporting Summary linked to this article.

## Supplementary information

SUPPLEMENTARY INFORMATION

Reporting Summary checklist

## Data Availability

The SPRINT data set which was used to create the risk models and decision recommendations can be made available through formal requests to the NHLBI. The CHS data set which was used to externally validate the models in this work cannot be made publicly available in a public depository due to patient confidentiality. Future requests for the CHS data used in this study, for the purpose of reproducing the study’s results, can be obtained via contacting Dr. Noa Dagan at noada@clalit.org.il. Requests for obtaining CHS data will have to be submitted and approved by the CHS Data Utilization and the CHS IRB before the data can be provided through secured virtual data sharing environments.

## Supplementary information

**Supplementary information** accompanies the paper on the *npj Digital Medicine* website (10.1038/s41746-019-0156-3).

## Code availability

Extraction codes that deal specifically with the Clalit Health Services internal database cannot be made accessible to the public due to the security of the internal databases. Since the codes used to produce the analysis of the SPRINT data include internal field names of the SPRINT data, they could be made available after receiving approval from the NHLBI Biologic Specimen and Data Repository Information Coordinating Centre for accessing the SPRINT data.
