# Supplementary material for: Translating clinical trial results into personalized recommendations by considering multiple outcomes and subjective views
Source: NPJ Digit Med. 2019 Aug 21;2:81. doi: 10.1038/s41746-019-0156-3 (PMC6704144; doi:10.1038/s41746-019-0156-3)
Supplement: Supplementary file 1 — SUPPLEMENTARY INFORMATION [file 41746_2019_156_MOESM1_ESM.pdf]

SUPPLEMENTAL MATERIAL

A process focused on practical usability for translating clinical trial results to personalized recommendations through a decision support interface

Noa Dagan, MD; Morton Leibowitz, MD; Meytal Avgil Tsadok, PhD; Chandra Cohen-Stavi, MPA; Moshe Hoshen, PhD; Tomas Karpati, MD; Amichay Akriv, MStat; Ilan Gofer; Harel Gilutz, MD; Eduardo Podjarny, MD; Eitan Bachmat, PhD; Ran Balicer, MD.

Table of Contents

Supplementary Table 1. Prediction models' selected variables 2

Supplementary Table 2. Performance of the prediction (discrimination and overall calibration) models over three year follow-up period 3

Supplementary Figure 1. Calibration plots for the SPRINT population (using final prediction models and out-of-sample models) and the Clalit Health Services population 4

Supplementary Table 3a. Calibration by deciles for the SPRINT population (using final prediction models and out-of-sample models) and the Clalit Health Services population: Main outcomes... 5

Supplementary Table 3b. Calibration tables by deciles for the SPRINT population (using final prediction models and out-of-sample models) and the CHS population: Major adverse events... 6

Supplementary Table 4. Severity weights by physicians 8

Supplementary Table 5. Population characteristics by recommendation groups 10

Supplementary Tables 6a and 6b Sensitivity analyses examining the recommendation distribution by severity weightings of each physician and the agreement of resulting recommendations 13

Supplementary Table 7. Additional analysis of treatment recommendation's validity 14

Supplementary Table 8. External validation using Clalit Health Services' data: Study population definition 15

Supplementary Description 1. The multiple imputation and variable selection process 16

Supplementary Table 9. External validation using Clalit Health Services' data: Extraction rules for variables 17

References 20

Supplementary Table 1. Prediction models' selected variables

Variables	Acute myocardial infarction: beta (95% CI)	Stroke: beta (95% CI)	Acute decompensated heart failure: beta (95% CI)	Cardiovascular death: beta (95% CI)	Serious hypotension: beta (95% CI)	Serious syncope: beta (95% CI)	Serious electrolyte abnormality: beta (95% CI)	Serious acute kidney injury: beta (95% CI)
Randomization variable (intensive treatment)	-0.187 (-0.450-0.077)	-0.108 (-0.461-0.246)	0.872 (-0.392-2.136)<br><br>-0.023* (-0.044--0.002)	-0.564 (-0.974--0.155)	0.516 (0.208-0.825)	0.287 (-0.011-0.584)	0.313 (0.068-0.558)	0.522 (0.283-0.760)
Age (years)	0.055 (0.039-0.071)	0.055 (0.035-0.075)	0.079 (0.055-0.102)	0.032 (0.002-0.061)	0.000 (0.000 - 0.000)	0.050 (0.033-0.067)	0.020 (0.006-0.035)	0.023 (0.009-0.036)
Sex - Female	0.000 (0.000 - 0.000)	0.000 (0.000 - 0.000)	0.000 (0.000 - 0.000)	-0.853 (-1.330--0.376)	-0.489 (-0.834--0.145)	0.000 (0.000 - 0.000)	0.663 (0.413-0.913)	-0.669 (-0.937--0.400)
Black race	0.000 (0.000 - 0.000)	0.000 (0.000 - 0.000)	0.000 (0.000 - 0.000)	0.000 (0.000 - 0.000)	0.000 (0.000 - 0.000)	0.000 (0.000 - 0.000)	0.000 (0.000 - 0.000)	0.648 (0.411-0.885)
BMI (mg/kg^2)	0.000 (0.000 - 0.000)	0.000 (0.000 - 0.000)	0.043 (0.014-0.073)	0.000 (0.000 - 0.000)	0.000 (0.000 - 0.000)	0.000 (0.000 - 0.000)	-0.046 (-0.074--0.017)	0.000 (0.000 - 0.000)
Smoking status – Former smoker	0.393 (0.081-0.706)	0.000 (0.000 - 0.000)	0.000 (0.000 - 0.000)	0.000 (0.000 - 0.000)	0.000 (0.000 - 0.000)	0.000 (0.000 - 0.000)	0.000 (0.000 - 0.000)	0.315 (0.057-0.573)
Smoking status – Current smoker	1.158 (0.735-1.582)	0.000 (0.000 - 0.000)	0.873 (0.387-1.359)	0.000 (0.000 - 0.000)	0.632 (0.253-1.012)	0.786 (0.377-1.195)	0.000 (0.000 - 0.000)	0.660 (0.305-1.016)
Clinical or sub-clinical CVD	0.755 (0.471-1.039)	0.000 (0.000 - 0.000)	0.575 (0.241-0.908)	0.785 (0.366-1.205)	0.486 (0.150-0.822)	0.000 (0.000 - 0.000)	0.000 (0.000 - 0.000)	0.308 (0.038-0.578)
Systolic blood pressure (mmHg)	0.000 (0.000 - 0.000)	0.016 (0.006-0.027)	0.000 (0.000 - 0.000)	0.016 (0.004-0.029)	0.000 (0.000 - 0.000)	0.000 (0.000 - 0.000)	0.010 (0.003-0.018)	0.011 (0.004-0.019)
Number of blood pressure medication types	0.000 (0.000 - 0.000)	0.000 (0.000 - 0.000)	0.327 (0.170-0.483)	0.294 (0.108-0.480)	0.181 (0.020-0.342)	0.000 (0.000 - 0.000)	0.320 (0.202-0.438)	0.162 (0.051-0.273)
eGFR (mL/min/1.73m^2)	0.000 (0.000 - 0.000)	0.000 (0.000 - 0.000)	-0.008 (-0.020-0.004)	-0.018 (-0.032--0.005)	-0.019 (-0.027--0.011)	0.000 (0.000 - 0.000)	0.000 (0.000 - 0.000)	-0.038 (-0.045--0.031)
Total Cholesterol (mg/dl)	0.005 (0.001-0.009)	0.000 (0.000 - 0.000)	0.000 (0.000 - 0.000)	0.000 (0.000 - 0.000)	0.000 (0.000 - 0.000)	-0.006 (-0.011--0.002)	0.000 (0.000 - 0.000)	0.000 (0.000 - 0.000)
HDL (mg/dl)	-0.022 (-0.034--0.009)	-0.017 (-0.034--0.001)	0.000 (0.000 - 0.000)	0.000 (0.000 - 0.000)	0.013 (0.003-0.023)	0.014 (0.004-0.024)	0.000 (0.000 - 0.000)	0.000 (0.000 - 0.000)

Abbreviations: CI, confidence interval; BMI, body mass index; eGFR, estimated glomerular filtration rate; CVD, cardiovascular disease; HDL, high-density lipoprotein.

* Interaction term between the randomization variable and the eGFR variable.

Supplementary Table 2. Performance of the prediction (discrimination and overall calibration) models over three year follow-up period

Cardiovascular outcomes of high blood pressure																
	Acute myocardial infarction				Cerebrovascular accident				Decompensated heart failure				Cardiovascular death			
**Discrimination**	AUC, % (95% CI)				AUC, % (95% CI)				AUC, % (95% CI)				AUC, % (95% CI)			
SPRINT final models	69.6 (65.8-73.4)				67.8 (62.5-73.2)				77.5 (73.3-81.8)				75.3 (70.2-80.5)			
SPRINT out-of-sample	68.3 (64.3-72.4)				66.5 (60.8-72.2)				76.4 (72.1-80.7)				73.7 (68.7-78.7)			
CHS external validation	67.1 (66.1-68.0)				65.3 (64.3-66.2)				75.5 (74.7-76.4)				NA			
**Calibration**	Event, N	Observed risk (%)	Mean predicted risk (%)	Observed/ Predicted ratio	Event, N	Observed risk (%)	Mean predicted risk (%)	Observed/ Predicted ratio	Event, N	Observed risk (%)	Mean predicted risk (%)	Observed/ Predicted ratio	Event, N	Observed risk (%)	Mean predicted risk (%)	Observed/ Predicted ratio
SPRINT final models	186	3.05	2.62	1.17	119	1.96	2.12	0.92	136	2.24	3.17	0.71	87	1.42	3.13	0.45
SPRINT out-of-sample	186	3.05	2.61	1.17	119	1.96	2.12	0.92	136	2.24	3.16	0.71	87	1.42	3.11	0.46
CHS external validation	3037	3.68	3.60	1.02	3330	4.03	2.72	1.48	3554	4.27	4.68	0.91	NA	NA	NA	NA

Adverse events of intensive blood pressure lowering																
	Serious hypotension				Serious syncope				Serious electrolyte abnormality				Serious acute kidney injury			
**Discrimination**	AUC, % (95% CI)				AUC, % (95% CI)				AUC, % (95% CI)				AUC, % (95% CI)			
SPRINT final models	68.1 (63.7-72.5)				69.0 (64.6-73.5)				68.8 (65.3-72.3)				77.2 (74.2-80.2)			
SPRINT out-of-sample	66.2 (61.7-70.8)				67.6 (63.3-72.0)				67.5 (64.0-71.0)				76.3 (73.2-79.4)			
CHS external validation	66.4 (64.1-68.7)				67.0 (66.0-68.1)				72.7 (71.8-73.5)				76.8 (75.8-77.7)			
**Calibration**	Event, N	Observed risk (%)	Mean predicted risk (%)	Observed/ Predicted ratio	Event, N	Observed risk (%)	Mean predicted risk (%)	Observed/ Predicted ratio	Event, N	Observed risk (%)	Mean predicted risk (%)	Observed/ Predicted ratio	Event, N	Observed risk (%)	Mean predicted risk (%)	Observed/ Predicted ratio
SPRINT final models	155	2.69	1.83	1.47	146	2.53	2.03	1.25	218	3.76	3.52	1.07	262	4.49	3.91	1.15
SPRINT out-of-sample	155	2.69	1.83	1.47	146	2.53	2.03	1.25	218	3.76	3.52	1.07	262	4.49	3.90	1.15
CHS external validation	539	0.66	1.36	0.48	2380	2.89	2.30	1.26	3348	4.02	3.75	1.07	2756	3.31	2.06	1.61

Abbreviations: AUC, area under the curve; CI, confidence interval; SPRINT, systolic blood pressure intervention trial; CHS, Clalit Health Services.

Supplementary Figure 1. Calibration plots for the SPRINT population (using final prediction models and out-of-sample models) and the Clalit Health Services population

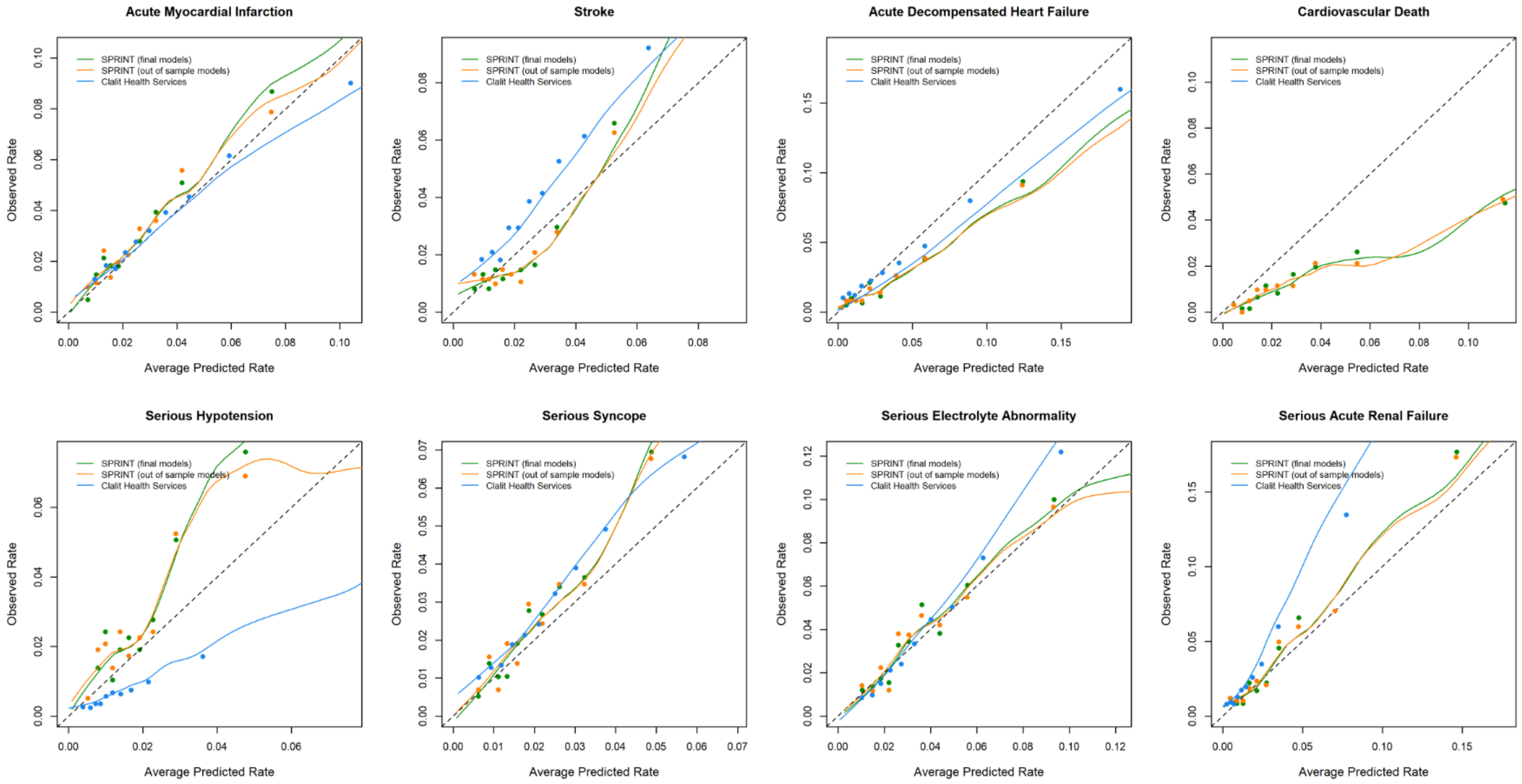

Abbreviations: SPRINT, Systolic Blood Pressure Intervention Trial.

Supplementary Table 3a. Calibration by deciles for the SPRINT population (using final prediction models and out-of-sample models) and the Clalit Health Services population: Main outcomes

	Acute myocardial infarction				Stroke				Acute decompensated heart failure				Cardiovascular death			
	Events	Observed risk (%)	Mean predicted risk (%)	Observed / Predicted ratio	Events	Observed risk (%)	Mean predicted risk (%)	Observed/ Predicted ratio	Events	Observed risk (%)	Mean predicted risk (%)	Observed/ Predicted ratio	Events	Observed risk (%)	Mean predicted risk (%)	Observed/ Predicted ratio
**SPRINT final models' analysis**																
Decile 1	3.0	0.49	0.71	0.69	5.0	0.82	0.69	1.19	2.0	0.33	0.25	1.30	2.0	0.33	0.45	0.73
Decile 2	9.0	1.48	1.03	1.43	8.0	1.32	0.97	1.36	3.0	0.49	0.56	0.88	1.0	0.16	0.79	0.21
Decile 3	13.0	2.13	1.30	1.64	5.0	0.82	1.17	0.70	6.0	0.99	0.88	1.12	1.0	0.16	1.09	0.15
Decile 4	11.2	1.84	1.56	1.18	9.0	1.48	1.38	1.08	5.0	0.82	1.21	0.68	4.0	0.65	1.41	0.47
Decile 5	11.0	1.80	1.84	0.98	7.0	1.16	1.62	0.71	4.0	0.66	1.63	0.40	7.0	1.15	1.76	0.65
Decile 6	13.8	2.26	2.19	1.03	8.0	1.32	1.89	0.70	13.0	2.14	2.14	1.00	5.0	0.82	2.23	0.37
Decile 7	17.0	2.79	2.63	1.06	9.0	1.48	2.21	0.67	7.0	1.15	2.85	0.40	10.0	1.64	2.87	0.57
Decile 8	24.0	3.93	3.22	1.22	10.0	1.65	2.66	0.62	16.0	2.63	3.91	0.67	12.0	1.97	3.78	0.52
Decile 9	31.0	5.09	4.18	1.22	18.0	2.97	3.39	0.88	23.0	3.78	5.82	0.65	16.0	2.62	5.47	0.48
Decile 10	53.0	8.69	7.50	1.16	40.0	6.59	5.26	1.25	57.0	9.38	12.42	0.75	29.0	4.75	11.47	0.41
**SPRINT out of sample analysis**																
Decile 1	6.0	0.98	0.71	1.39	8.0	1.32	0.69	1.91	2.0	0.33	0.25	1.31	2.0	0.33	0.44	0.75
Decile 2	7.0	1.15	1.03	1.12	7.0	1.15	0.97	1.19	5.0	0.82	0.56	1.48	0.0	0.00	0.78	0.00
Decile 3	14.8	2.43	1.30	1.87	7.0	1.15	1.17	0.99	5.0	0.82	0.87	0.94	3.0	0.49	1.09	0.45
Decile 4	8.4	1.38	1.56	0.88	6.0	0.99	1.38	0.72	5.0	0.82	1.20	0.68	6.0	0.98	1.40	0.70
Decile 5	12.0	1.97	1.84	1.07	9.0	1.49	1.62	0.92	5.0	0.82	1.62	0.51	6.0	0.98	1.76	0.56
Decile 6	13.8	2.26	2.18	1.04	8.0	1.32	1.89	0.70	10.4	1.71	2.13	0.80	7.0	1.15	2.21	0.52
Decile 7	20.0	3.28	2.62	1.25	6.4	1.05	2.20	0.48	8.6	1.41	2.84	0.50	7.0	1.15	2.85	0.40
Decile 8	22.0	3.61	3.22	1.12	12.6	2.08	2.66	0.78	16.0	2.63	3.91	0.67	13.0	2.13	3.77	0.56
Decile 9	34.0	5.58	4.18	1.34	17.0	2.80	3.39	0.83	23.6	3.88	5.83	0.67	13.0	2.13	5.46	0.39
Decile 10	48.0	7.87	7.47	1.05	38.0	6.26	5.26	1.19	55.4	9.11	12.38	0.74	30.0	4.91	11.38	0.43
**CHS external validation of final models**																
Decile 1	107.2	1.30	0.96	1.35	152.0	1.84	0.93	1.97	85.2	1.02	0.35	2.96	NA	NA	NA	NA
Decile 2	151.6	1.84	1.38	1.33	173.0	2.09	1.27	1.64	110.6	1.33	0.75	1.78	NA	NA	NA	NA

Decile 3	141.0	1.71	1.73	0.99	150.0	1.81	1.54	1.18	100.4	1.21	1.12	1.07	NA	NA	NA	NA
Decile 4	194.0	2.35	2.10	1.12	243.4	2.94	1.81	1.62	155.0	1.86	1.59	1.18	NA	NA	NA	NA
Decile 5	229.0	2.78	2.49	1.11	243.8	2.95	2.12	1.39	186.2	2.24	2.19	1.02	NA	NA	NA	NA
Decile 6	264.8	3.21	2.97	1.08	319.6	3.86	2.48	1.56	234.4	2.82	2.99	0.94	NA	NA	NA	NA
Decile 7	323.6	3.92	3.58	1.10	343.0	4.15	2.91	1.43	293.0	3.52	4.11	0.86	NA	NA	NA	NA
Decile 8	374.4	4.54	4.44	1.02	435.2	5.26	3.45	1.52	394.6	4.75	5.83	0.81	NA	NA	NA	NA
Decile 9	507.4	6.15	5.93	1.04	508.0	6.14	4.28	1.43	664.2	7.99	8.88	0.90	NA	NA	NA	NA
Decile 10	744.0	9.02	10.40	0.87	762.0	9.21	6.37	1.45	1330.4	16.00	18.95	0.84	NA	NA	NA	NA

Abbreviations: SPRINT, Systolic Blood Pressure Intervention Trial; CHS, Clalit Health Services.

Supplementary Table 3b. Calibration tables by deciles for the SPRINT population (using final prediction models and out-of-sample models) and the CHS population: Major adverse events

	Serious hypotension				Serious syncope				Serious electrolyte abnormality				Serious acute kidney injury			
	Events	Observed risk (%)	Mean predicted risk (%)	Observed / Predicted ratio	Events	Observed risk (%)	Mean predicted risk (%)	Observed/ Predicted ratio	Events	Observed risk (%)	Mean predicted risk (%)	Observed/ Predicted ratio	Events	Observed risk (%)	Mean predicted risk (%)	Observed/ Predicted ratio
**SPRINT final models' analysis**																
Decile 1	3.0	0.52	0.52	0.99	3.0	0.52	0.62	0.84	7.0	1.21	1.04	1.16	7.0	1.20	0.47	2.56
Decile 2	8.0	1.39	0.79	1.76	8.0	1.39	0.89	1.56	8.0	1.38	1.50	0.92	5.0	0.86	0.89	0.96
Decile 3	14.0	2.43	0.99	2.45	6.0	1.04	1.11	0.94	10.0	1.72	1.85	0.93	5.0	0.86	1.26	0.68
Decile 4	6.0	1.04	1.18	0.88	6.0	1.04	1.33	0.78	9.0	1.55	2.20	0.70	13.0	2.23	1.67	1.34
Decile 5	11.0	1.91	1.39	1.37	11.0	1.91	1.58	1.21	19.0	3.28	2.62	1.25	10.0	1.71	2.14	0.80
Decile 6	13.0	2.26	1.62	1.39	16.0	2.78	1.86	1.49	20.0	3.45	3.06	1.13	13.0	2.23	2.73	0.82
Decile 7	11.0	1.91	1.92	1.00	15.4	2.67	2.19	1.22	29.8	5.14	3.62	1.42	26.6	4.56	3.52	1.30
Decile 8	16.0	2.77	2.27	1.22	19.6	3.40	2.62	1.30	22.2	3.83	4.40	0.87	38.4	6.59	4.76	1.38
Decile 9	29.2	5.07	2.89	1.75	21.0	3.65	3.23	1.13	35.0	6.04	5.59	1.08	41.0	7.03	7.02	1.00
Decile 10	43.8	7.59	4.76	1.59	40.0	6.94	4.88	1.42	58.0	10.00	9.33	1.07	103.0	17.67	14.65	1.21
**SPRINT out of sample analysis**																
Decile 1	3.0	0.52	0.52	0.99	4.0	0.69	0.62	1.12	8.2	1.41	1.04	1.36	7.0	1.20	0.47	2.56

Decile 2	11.0	1.91	0.79	2.42	9.0	1.56	0.89	1.76	6.8	1.17	1.50	0.79	6.0	1.03	0.89	1.16
Decile 3	12.0	2.08	0.99	2.10	4.0	0.69	1.11	0.63	13.0	2.24	1.85	1.21	6.0	1.03	1.26	0.81
Decile 4	8.0	1.39	1.18	1.17	11.0	1.91	1.33	1.44	7.0	1.21	2.20	0.55	11.0	1.89	1.67	1.13
Decile 5	14.0	2.43	1.39	1.75	8.0	1.39	1.58	0.88	22.0	3.80	2.61	1.45	13.8	2.36	2.13	1.11
Decile 6	10.0	1.74	1.62	1.07	17.0	2.95	1.86	1.59	21.8	3.76	3.06	1.23	12.2	2.09	2.73	0.77
Decile 7	13.0	2.25	1.91	1.18	14.0	2.43	2.19	1.11	27.0	4.66	3.62	1.29	29.0	4.97	3.52	1.41
Decile 8	14.0	2.43	2.27	1.07	20.0	3.47	2.61	1.33	24.4	4.21	4.40	0.96	35.0	6.00	4.75	1.26
Decile 9	30.2	5.24	2.89	1.81	20.0	3.47	3.23	1.08	31.8	5.49	5.58	0.98	41.0	7.03	7.02	1.00
Decile 10	39.8	6.90	4.75	1.45	39.0	6.77	4.87	1.39	56.0	9.66	9.32	1.04	101.0	17.32	14.61	1.19
**CHS external validation of final models**																
Decile 1	22.0	0.27	0.39	0.68	84.0	1.02	0.63	1.62	72.0	0.87	1.03	0.84	67.2	0.81	0.26	3.16
Decile 2	20.0	0.24	0.59	0.41	105.0	1.28	0.93	1.37	81.0	0.97	1.48	0.66	78.6	0.94	0.50	1.88
Decile 3	29.0	0.35	0.73	0.48	110.0	1.34	1.18	1.13	125.0	1.50	1.86	0.81	67.0	0.80	0.71	1.13
Decile 4	29.2	0.36	0.86	0.41	155.0	1.88	1.45	1.30	177.2	2.13	2.26	0.94	106.6	1.28	0.93	1.38
Decile 5	47.2	0.57	1.01	0.57	175.8	2.14	1.76	1.21	200.0	2.40	2.74	0.88	146.2	1.76	1.17	1.50
Decile 6	55.4	0.67	1.19	0.57	198.6	2.41	2.11	1.14	278.0	3.34	3.30	1.01	164.2	1.97	1.47	1.34
Decile 7	52.4	0.64	1.40	0.45	265.0	3.22	2.51	1.28	372.4	4.48	4.01	1.12	215.2	2.59	1.86	1.39
Decile 8	61.6	0.75	1.69	0.44	321.0	3.90	3.02	1.29	419.0	5.03	4.93	1.02	289.8	3.48	2.44	1.43
Decile 9	81.2	0.99	2.15	0.46	404.6	4.92	3.75	1.31	608.8	7.32	6.27	1.17	499.6	6.00	3.48	1.72
Decile 10	141.0	1.72	3.62	0.47	561.0	6.81	5.69	1.20	1014.6	12.19	9.64	1.26	1121.6	13.47	7.74	1.74

Abbreviations: SPRINT, Systolic Blood Pressure Intervention Trial; CHS, Clalit Health Services.

Supplementary Table 4. Severity weights by physicians

	Physician 1	Physician 2	Physician 3	Physician 4	Physician 5	Physician 6
Acute myocardial infarction	8	8	9	7	8	7
Stroke	9	8.5	9	7	8	8
Acute decompensated heart failure	7	8	9	7	8	6
Cardiovascular death	10	10	10	10	10	10
Serious hypotension	6	6	6	8	6.5	7
Serious syncope	7	7	7	7	7	9
Serious electrolyte abnormalities	6	6	7	7	6	8
Serious acute kidney injury	8	8	6	7	8	8
	**Physician 7**	**Physician 8**	**Physician 9**	**Physician 10**	**Physician 11**	**Physician 12**
Acute myocardial infarction	8	10	6	8	9	7
Stroke	8	10	8	9	9	9
Acute decompensated heart failure	8	8	8	9	9	8
Cardiovascular death	10	10	10	10	10	10
Serious hypotension	6	5	9	8	7	3
Serious syncope	6	7	6	8	9	3
Serious electrolyte abnormalities	6	6	8	8	8	6
Serious acute kidney injury	6	8	7	9	7	4
	**Average**	**Standard deviation**				
Acute myocardial infarction	7.9	1.08				
Stroke	8.5	0.78				
Acute decompensated heart failure	7.9	0.90				
Cardiovascular death	10.0	0.00				
Serious hypotension	6.5	1.56				
Serious syncope	6.9	1.56				
Serious electrolyte abnormalities	6.8	0.94				
Serious acute kidney injury	7.2	1.34				

Prior to providing severity weights, the physicians read the following definition by which SPRINT's adverse events were classified as serious or not: "Serious adverse-events and their dates were recorded in cases of fatal or life-threatening event that resulted in clinically significant or persistent disability, that required or prolonged a hospitalization, or that were judged by the investigator to represent a clinically significant hazard or harm to the participant that might require medical intervention".

Supplementary Table 5. Population characteristics by recommendation groups

	SPRINT population			CHS population		
	Study population	Intensive recommendation		Study population	Intensive recommendation	
		No	Yes		No	Yes
**Characteristics**[*]	n=9,360	n=3,545 (37.9%)	n=5,815 (62.1%)	n=88,374	n=13,841 (15.7%)	n=74533 (84.3%)
Age, years						
50-59, n (%)	1,962 (21.0)	1137 (32.1)	825 (14.2)	13,162 (14.9)	3961 (28.6)	9201 (12.3)
60-69, n (%)	3,409 (36.4)	1348 (38.0)	2061 (35.4)	25,634 (29.0)	4546 (32.8)	21088 (28.3)
70-79, n (%)	2,823 (30.2)	783 (22.1)	2040 (35.1)	26,210 (29.7)	3143 (22.7)	23067 (30.9)
80-89, n (%)	1,107 (11.8)	263 (7.4)	844 (14.5)	19,593 (22.2)	1856 (13.4)	17737 (23.8)
90-99, n (%)	59 (0.6)	14 (0.4)	45 (0.8)	3,775 (4.3)	335 (2.4)	3440 (4.6)
Missing (%)	0 (0.0)	0 (0.0)	0 (0.0)	0 (0.0)	0 (0.0)	0 (0.0)
Mean ± SD	67.9 ± 9.4	64.9 ± 9.2	69.7 ± 9.1	71.7 ± 10.7	67.2 ± 10.8	72.5 ± 10.5
Sex						
Female, n (%)	3,331 (35.6)	1805 (50.9)	1526 (26.2)	41,713 (47.2)	8642 (62.4)	33071 (44.4)
Male, n (%)	6,029 (64.4)	1740 (49.1)	4289 (73.8)	46,661 (52.8)	5199 (37.6)	41462 (55.6)
Missing (%)	0 (0.0)	0 (0.0)	0 (0.0)	0 (0.0)	0 (0.0)	0 (0.0)
Black race						
No, n (%)	6,414 (68.5)	1888 (53.3)	4526 (77.8)	87,456 (99.0)	13625 (98.4)	73831 (99.1)
Yes, n (%)	2,946 (31.5)	1657 (46.7)	1289 (22.2)	918 (1.0)	216 (1.6)	702 (0.9)
Missing	0 (0.0)	0 (0.0)	0 (0.0)	0 (0.0)	0 (0.0)	0 (0.0)
BMI[†], kg/m^2						
Underweight, n (%)	47 (0.5)	35 (1.0)	12 (0.2)	660 (0.7)	271 (2.0)	389 (0.5)
Normal, n (%)	1,682 (18.0)	931 (26.3)	751 (12.9)	20,797 (23.5)	5450 (39.4)	15347 (20.6)
Overweight, n (%)	3,599 (38.5)	1413 (39.9)	2186 (37.6)	38,257 (43.3)	5777 (41.7)	32480 (43.6)
Obese, n (%)	3,955 (42.3)	1131 (31.9)	2824 (48.6)	28,400 (32.1)	2305 (16.7)	26095 (35.0)
Missing (%)	77 (0.8)	35 (1.0)	42 (0.7)	260 (0.3)	38 (0.3)	222 (0.3)
Mean ± SD	29.9 ± 5.8	28.3 ± 5.3	30.8 ± 5.8	28.4 ± 5.0	26.2 ± 4.4	28.8 ± 5.0
Smoking category						
Never, n (%)	4,122 (44.0)	1605 (45.3)	2517 (43.3)	56,790 (64.3)	8573 (61.9)	48217 (64.7)
Former, n (%)	3,973 (42.4)	1322 (37.3)	2651 (45.6)	17,472 (19.8)	2419 (17.5)	15053 (20.2)
Current, n (%)	1,239 (13.2)	602 (17.0)	637 (11.0)	13,166 (14.9)	2757 (19.9)	10409 (14.0)
Missing (%)	26 (0.3)	16 (0.5)	10 (0.2)	946 (1.1)	92 (0.7)	854 (1.1)

	SPRINT population			CHS population		
		Intensive recommendation			Intensive recommendation	
	Study population	No	Yes	Study population	No	Yes
Clinical/subclinical CVD						
No, n (%)	7,483 (79.9)	3288 (92.8)	4195 (72.1)	58,526 (66.2)	12465 (90.1)	46061 (61.8)
Yes, n (%)	1,877 (20.1)	257 (7.2)	1620 (27.9)	29,848 (33.8)	1376 (9.9)	28472 (38.2)
Missing (%)	0 (0.0)	0 (0.0)	0 (0.0)	0 (0.0)	0 (0.0)	0 (0.0)
Systolic BP, mmHg						
<120, n (%)	754 (8.1)	310 (8.7)	444 (7.6)	0 (0.0)	0 (0.0)	0 (0.0)
120-139, n (%)	4,210 (45.0)	1592 (44.9)	2618 (45.0)	46,228 (52.3)	6978 (50.4)	39250 (52.7)
140-159, n (%)	3,420 (36.5)	1273 (35.9)	2147 (36.9)	36,309 (41.1)	5701 (41.2)	30608 (41.1)
160-179, n (%)	846 (9.0)	323 (9.1)	523 (9.0)	5,575 (6.3)	1091 (7.9)	4484 (6.0)
≥180, n (%)	130 (1.4)	47 (1.3)	83 (1.4)	262 (0.3)	71 (0.5)	191 (0.3)
Missing (%)	0 (0.0)	0 (0.0)	0 (0.0)	0 (0.0)	0 (0.0)	0 (0.0)
Mean ± SD	139.7 ± 15.6	139.2 ± 15.9	139.9 ± 15.4	139.9 ± 10.0	140.6 ± 10.7	139.8 ± 9.9
BP medication types						
0-1, n (%)	3,635 (38.8)	1520 (42.9)	2115 (36.4)	42,576 (48.2)	8021 (58.0)	34555 (46.4)
2-3, n (%)	5,211 (55.7)	1857 (52.4)	3354 (57.7)	41,842 (47.3)	5408 (39.1)	36434 (48.9)
≥4, n (%)	514 (5.5)	168 (4.7)	346 (6.0)	3,956 (4.5)	412 (3.0)	3544 (4.8)
Missing (%)	0 (0.0)	0 (0.0)	0 (0.0)	0 (0.0)	0 (0.0)	0 (0.0)
eGFR, mL/min/1·73m^2						
<20, n (%)	14 (0.1)	14 (0.4)	0 (0.0)	0 (0.0)	0 (0.0)	0 (0.0)
20-39, n (%)	524 (5.6)	488 (13.8)	36 (0.6)	2,197 (2.5)	1755 (12.7)	442 (0.6)
40-59, n (%)	2,112 (22.6)	1142 (32.2)	970 (16.7)	12,896 (14.6)	4284 (31.0)	8612 (11.6)
60-79, n (%)	3,632 (38.8)	1147 (32.4)	2485 (42.7)	34,694 (39.3)	4648 (33.6)	30046 (40.3)
80-99, n (%)	2,269 (24.2)	542 (15.3)	1727 (29.7)	26,646 (30.2)	2192 (15.8)	24454 (32.8)
≥100, n (%)	772 (8.2)	190 (5.4)	582 (10.0)	11,814 (13.4)	944 (6.8)	10870 (14.6)
Missing (%)	37 (0.4)	22 (0.6)	15 (0.3)	127 (0.1)	18 (0.1)	109 (0.1)
Mean ± SD	71.7 ± 20.6	63.3 ± 21.8	76.9 ± 18.0	78.2 ± 21.1	65.7 ± 25.1	80.6 ± 19.4
Total cholesterol, mg/dL						
<200, n (%)	5,794 (61.9)	1959 (55.3)	3835 (66.0)	57,926 (65.5)	7932 (57.3)	49994 (67.1)
200-239, n (%)	2,480 (26.5)	1093 (30.8)	1387 (23.9)	22,114 (25.0)	4156 (30.0)	17958 (24.1)
≥240, n (%)	1,048 (11.2)	469 (13.2)	579 (10.0)	8,200 (9.3)	1734 (12.5)	6466 (8.7)
Missing (%)	38 (0.4)	24 (0.7)	14 (0.2)	134 (0.2)	19 (0.1)	115 (0.2)
Mean ± SD	190.1 ± 41.2	196.7 ± 39.9	186.1 ± 41.4	188.0 ± 38.3	196.5 ± 37.9	186.4 ± 38.1
HDL, mg/dL						
<40, n (%)	1,418 (15.1)	354 (10.0)	1064 (18.3)	18,380 (20.8)	1777 (12.8)	16603 (22.3)
40-59, n (%)	5,409 (57.8)	1809 (51.0)	3600 (61.9)	50,589 (57.2)	7214 (52.1)	43375 (58.2)
≥60, n (%)	2,495 (26.7)	1358 (38.3)	1137 (19.6)	19,259 (21.8)	4829 (34.9)	14430 (19.4)
Missing (%)	38 (0.4)	24 (0.7)	14 (0.2)	146 (0.2)	21 (0.2)	125 (0.2)
Mean ± SD	52.9 ± 14.5	57.6 ± 16.5	50.0 ± 12.2	50.4 ± 13.4	55.9 ± 15.8	49.4 ± 12.7

Abbreviations: SPRINT, systolic blood pressure intervention trial; CHS, Clalit health services; SD, standard deviation; BMI, body mass index; CVD, cardiovascular disease; BP blood pressure; eGFR, estimated glomerular filtration rate; HDL, high-density lipoprotein; SD, standard deviation.

[*]Variables were taken from the baseline characteristics of the SPRINT population and from the available EHR information prior to January 1st, 2013 for the CHS population.

[†]Body mass index was measured as the weight in kilograms divided by the square of the height in meters.

Supplementary Tables 6a and 6b Sensitivity analyses examining the recommendation distribution by severity weightings of each physician and the agreement of resulting recommendations

Table S8a. Recommendation distribution according to severity weightings of each physician

	Physician 1	Physician 2	Physician 3	Physician 4	Physician 5	Physician 6
**No recommendation**	40.3%	36.8%	27.0%	47.6%	38.5%	60.2%
**Yes recommendation**	59.7%	63.2%	73.0%	52.4%	61.5%	39.8%
	**Physician 7**	**Physician 8**	**Physician 9**	**Physician 10**	**Physician 11**	**Physician 12**
**No recommendation**	26.9%	31.1%	48.3%	49.3%	39.7%	10.1%
**Yes recommendation**	73.1%	68.8%	51.7%	50.6%	60.3%	89.9%

Table S8b. Proportion of patients for whom there was agreement in the resulting recommendation across physicians' severity weightings

	No	Yes	Proportion of patients	Benefit-harm ratio (average)
**Number of physicians whose severity weights yielded patients in the "No treatment" or "Yes treatment" groups**	12	0	10.1%	0.46
	11	1	16.2%	0.75
	10	2	1.1%	0.86
	9	3	4.2%	0.90
	8	4	4.8%	0.95
	7	5	1.6%	0.99
	6	6	1.6%	1.00
	5	7	2.0%	1.03
	4	8	5.0%	1.08
	3	9	1.7%	1.12
	2	10	2.4%	1.14
	1	11	9.9%	1.24
	0	12	39.6%	1.85

Supplementary Table 7. Additional analysis of treatment recommendation's validity

Treatment recommendation testing: evaluation of the SPRINT outcomes within each recommendation group using the out-of-sample models

	**Original SPRINT randomization**		**Entire SPRINT population**	**Recommendation group: Intensive treatment**	**Recommendation group: Non-intensive treatment**
Number of participants	Actual intensive		4,677	2,916	1,761
	Actual non-intensive		4,683	2,916	1,767
Average systolic blood pressure during follow-up* (mmHg)	Actual intensive		121.01	120.73	121.46
	Actual non-intensive		135.28	135.01	135.73
Primary composite cardiovascular outcome (% per year)	Actual intensive		1.65	1.78	1.45
	Actual non-intensive		2.19	2.54	1.63
		Ratio (95% CI)	0.75 (0.64-0.89)	0.70 (0.57-0.86)	0.89 (0.66-1.19)
		P-value	0.00092	0.00054	0.42636
All-cause mortality (% per year)	Actual intensive		1.03	1.01	1.05
	Actual non-intensive		1.40	1.53	1.18
		Ratio (95% CI)	0.74 (0.60-0.91)	0.66 (0.51-0.86)	0.89 (0.63-1.26)
		P-value	0.00355	0.00169	0.51211
Severe adverse event composite outcome (% per year)	Actual intensive		2.89	2.56	3.42
	Actual non-intensive		2.03	1.83	2.34
		Ratio (95% CI)	1.42 (1.22-1.65)	1.40 (1.14-1.71)	1.46 (1.16-1.83)
		P-value	<0.00001	0.00112	0.00103

Abbreviations: SPRINT, Systolic Blood Pressure Intervention Trial; mmHg; CI, confidence interval.
*Using the last three measurements of each participant.

Supplementary Table 8. External validation using Clalit Health Services' data: Study population definition

Candidates for the study population were Clalit Health Services (CHS) members who were older than 50 years as of the index date (January 1st, 2013) and had continuous CHS membership in the year prior to the index date through the end of 2015 (or date of death, whichever came first).

Inclusion Criteria	Exclusion Criteria
Hypertension (see details in Table S3)	Diagnosis of diabetes mellitus
Age over 50 years	Had a prior stroke
Last systolic blood pressure of:<br>- 130 – 180 mmHg if on 0 or 1 medication<br>- 130 – 170 mmHg if on up to 2 medications<br>- 130 – 160 mmHg if on up to 3 medications<br>- 130 – 150 mmHg if on up to 4 medications	Diagnosis of polycystic kidney disease
Increased cardiovascular risk, defined by one or more of the following:<br>- Clinical or subclinical cardiovascular disease (CVD) (see Table S3), other than stroke<br>- Chronic Kidney Disease with an estimated glomerular filtration rate (eGFR) of 20 ml/min/1.73m^2 to less than 60 ml/min/1.73m^2, calculated using the modification of diet in renal disease (MDRD) formula<br>- 10-year risk of CVD of 15% or greater, based on the Framingham risk score<br>- Age of 75 years or older	eGFR < 20 ml/min/1.73m^2 or end-stage renal disease (dialysis)
	Having had a cardiovascular event, procedure, or hospitalization for unstable angina within last 3 months
	Had any organ transplant
	Pregnant during the follow-up period

Exclusion criteria that were used in the systolic blood pressure intervention (SPRINT) trial but not listed above were not used because they could not be defined in the CHS electronic health record data:

- Known secondary cause of hypertension that causes concern regarding safety of the protocol
- One minute standing SBP < 110 mm Hg.
- Proteinuria
- Arm circumference too large or small to allow accurate blood pressure measurement
- left ventricular ejection fraction (by any method) < 35%
- A medical condition likely to limit survival to less than 3 years
- Any factors judged by the clinic team to be likely to limit adherence to interventions
- 19. Unintentional weight loss > 10% in last 6 months

Supplementary Description 1. The multiple imputation and variable selection process

Multiple imputation of each missing variable was done based on the other predictors, outcome indicators (yes/no event), and time stamps (both as original time and log time) as recommended for cases of survival data

The variable selection process was carried out using backwards selection. The initial model included the trial's randomisation (intensive treatment) variable, the 11 potential predictors, and their interaction with the intensive treatment variable. Quantitative variables were included in the models as continuous variables. As suggested by Van Buuren,[1] variable selection for each model was done in two phases. In the first phase, variables were selected separately in each imputed dataset using backwards selection based on the Bayesian Information Criterion (BIC).[2]

Then, based on majority vote, variables that were chosen in more than half of the imputed datasets comprised the candidates for the second phase, in which backwards selection using the Wald statistic (calculated from the multiply imputed data) was employed to determine the final predictors for each model.[1] In both phases, the intensive treatment variable was resistant to elimination. Quantitative variables were included in the models as continuous variables.

Supplementary Table 9. External validation using Clalit Health Services' data: Extraction rules for variables

The following table describes the method in which different variables were defined using the Clalit Health Services' (CHS) electronic health records.
Diagnostic codes were extracted both from the community setting and admission diagnoses, as well as from the CHS chronic condition registry. Codes that were recorded from the community setting were also further validated using physicians' free text notes (which only exist in these records). In cases in which the listed codes seemed too broad for the relevant diagnoses, further narrowing was performed using the free text phrases.
Laboratory test results were extracted using internal codes.
Medication use information was extracted based on comprehensive CHS purchase records using the Anatomical Therapeutic Chemical (ATC) classification system.

Variable	Variable type	Relevant Extraction Rules*
Hypertension	Basic cohort characteristic	Diagnosis codes:<br>ICD-9 codes: 40[12345]%<br>ICPC codes: K86<br>Additionally, relevant codes from the CHS chronic condition registry were used.<br>Blood pressure measurements:<br>In addition to a diagnostic code, individuals in the study population were required to have a documentation of at least two blood pressure measurements in the hypertensive range, no more than two years apart.
Acute myocardial infarction	Model outcome	ICD-9 codes: 410%<br>Extracted only from primary or secondary diagnoses of admissions during the follow-up period<br>OR ICD-9 codes: 410%<br>Extracted from community records given accompany text suggesting acute exacerbation
Stroke	Model outcome	ICD-9 codes: 43[016]%; 43[34]._1%; 437.1%<br>Extracted only from primary or secondary diagnoses of admissions during the follow-up period<br>OR ICD-9 codes: 43[016]%; 43[34]._1%; 437.1%<br>Extracted from community records given accompany text suggesting acute exacerbation
Acute decompensated heart failure	Model outcome	ICD-9 codes: 428%<br>Extracted from primary or secondary diagnoses of admissions during the follow-up period<br>OR ICD-9 codes: 428%<br>Extracted from community records given accompany text suggesting acute exacerbation
Serious hypotension	Model outcome	ICD-9 codes: 458; 458.[^2]%; 458.2; 458.29; 796.3%; 785.5; 785.5[09]<br>Extracted only from primary or secondary diagnoses of admissions during the follow-up period
Serious syncope	Model outcome	ICD-9 codes: 780.2%<br>Extracted only from primary or secondary diagnoses of admissions during the follow-up period
Serious electrolyte abnormality	Model outcome	ICD-9 codes: 276; 276.[01234789]%<br>Extracted only from primary or secondary diagnoses of admissions during the follow-up period
Serious acute renal failure	Model outcome	ICD-9 codes: 584%<br>Extracted only from primary or secondary diagnoses of admissions during the follow-up period
Age	Model input	Calculated age based on date of birth

Sex	Model input	The documented sex within the CHS demography table
Black race	Model input	Individual's, parents' or grandparents' birth country documented to be Ethiopia (almost exclusive ethnicity of black race individuals in Israel)
BMI	Model input	Calculated based on last documented weight (kilograms), divided by the square of the height (meters)
Smoking status	Model input	Last documented smoking status
Clinical or subclinical cardiovascular disease	Model input	Any ischemic heart disease:<br>ICD-9 codes: 410%; 411%; 412%; 413%; 414.[023489]%<br>ICPC codes: K7[56]<br>Additionally, relevant codes from the CHS chronic condition registry were used.<br>OR Peripheral artery disease:<br>ICD-9 codes: 40%; 444%; 445%<br>ICPC codes: K92<br>OR Abdominal artery aneurysm:<br>ICD-9 codes: 441.[3467]%<br>OR Left ventricular hypertrophy:<br>ICD-9 codes: 429.3%<br>OR Percutaneous coronary intervention:<br>ICD-9 codes: V45.82;36.0%<br>Additionally, internal codes that are used to document medical procedures were used.<br>OR Coronary artery bypass graft:<br>ICD-9 codes: 36.1%; 414.04<br>Additionally, internal codes that are used to document medical procedures were used.<br>OR Carotid endarterectomy:<br>ICD-9 codes: 38.2%; 00.63; 39.22<br>Additionally, internal codes that are used to document medical procedures were used.<br><br>Parameters that were not included in the definition:<br>ECG changes on a graded exercise test (GXT), or positive cardiac imaging study;<br>At least a 50% diameter stenosis of a coronary, carotid, or lower extremity artery;<br>Coronary artery calcium score ≥ 400 Agatston units within the past 2 years;<br>Ankle brachial index (ABI) ≤0.90 within the past 2 years;<br>Left ventricular hypertrophy (LVH) by ECG;
Systolic blood pressure	Model input	Last documented systolic blood pressure
Number of blood pressure lowering medications types	Model input	Based on purchase records of medications within the 6 months prior to the index date: (number of distinct ATC3 codes in the format of C0[23]%) + (number of distinct ATC2 codes in the format of C0[789]%)
eGFR	Model input	Calculated according to the last documentation of a creatinine test using the modification of diet in renal disease (MDRD) formula
Total cholesterol	Model input	Last documented lab result
High density cholesterol	Model input	Last documented lab result

ICD-9, International Classification of Diseases, Ninth Revision; ICPC, International Classification of Primary Care; CHS, Clalit Health Services; BMI, body mass index; ATC, anatomical therapeutic chemical; eGFR, estimated glomerular filtration rate.

*There are three shorthand methods used for coding:

1. codes marked with %: the % is used to extract all sub-codes of the higher-level code. The % represents a place in the code which can be substituted by any number of characters (for example, 820% will be used to extract 820.1…820.9)
2. Codes marked with [ ]: The square brackets represent a single digit in the code which can be substituted by any one of the included digits (for example, the code 733.[01] will be used to extract both codes 733.0 and 733.1)
3. Codes marked with _: The underscore represents a single character in the code which can be substituted by any character.

References

1. S VB. *Flexible Imputation of Missing Data.* Boca Raton, FL: CRC Press, Amazon, 2012.
2. Hastie T, Tibshirani, R., Friedman, J. *The Elements of Statistical Learning: Data Mining, Inference, and Prediction*. Second Edition ed. New York, NY, USA: Springer Science+Business Media, LLC, 2009.
